# Genome-sequenced bacterial collection from sorghum aerial root mucilage

**DOI:** 10.1128/MRA.00468-23

**Published:** 2023-11-01

**Authors:** Marco E. Mechan-Llontop, John Mullet, Ashley Shade

**Affiliations:** 1 Great Lakes Bioenergy Research Center, Michigan State University, East Lansing, Michigan, USA; 2 Department of Biochemistry & Biophysics, Texas A&M University, College Station, Texas, USA; 3 Centre National de la Recherche Scientifique, Laboratoire d'Ecologie Microbienne (UMR CNRS 5557, UMR INRAE 1418, VetAgro Sup), Universite Claude Bernard Lyon 1, Villeurbanne, France; University of Arizona, Tucson, Arizona, USA

**Keywords:** plant microbiome, host-microbiota, genomes, bioenergy, agroecosystems, phyllosphere, diazotrophs, exudates

## Abstract

A collection of 47 bacteria isolated from the mucilage of aerial roots of energy sorghum is available at the Great Lakes Bioenergy Research Center, Michigan State University, Michigan, USA. We enriched bacteria with putative plant-beneficial phenotypes and included information on phenotypic diversity, taxonomy, and whole genome sequences.

## ANNOUNCEMENT

Plant phyllosphere exudates harbor microbial communities that can engage with the host plant, for example, by supporting plant growth or resilience to various stresses (1). The heat- and drought-tolerant crop bioenergy sorghum (*Sorghum bicolor* L. Moench) secretes abundant polysaccharide-rich mucilage on aerial roots (2), which selects for specialized microbiome members that are expected to have plant-beneficial functions (3). As a resource for the genomic and functional diversities of bacteria associated with phyllosphere exudates, we present a collection of 47 bacterial strains isolated from sorghum aerial root mucilage. These strains represent 17 families and include 21 genera with putative plant-beneficial capabilities, including nitrogen fixation, phosphate solubilization, resistance to terpenoids, use of methanol as a carbon source, and tolerance to desiccation, based on the culture media in which the strains were originally isolated.

Bacterial strains were enriched from the mucilage of aerial roots of bioenergy sorghum cultivar TAM 17651 grown at the Great Lakes Bioenergy Research Center, as part of the Biofuel Cropping System Experiment in Hickory Corners, Michigan (42°23′41.6″ N, 85°22′23.1″ W). Aerial roots were harvested from sorghum plants grown on nitrogen-fertilized plots on 25 September 2020. We used sterile razor blades to collect between 3 and 5 aerial roots per plant into sterile 50 mL Eppendorf tubes. Within 2 hours of collection, the samples were transported to the laboratory on ice and stored at –80°C for 18 days until processing. Sorghum mucilage was first fully hydrated with sterile water, removed from roots, and then collected into sterile 1.5 mL Eppendorf tubes. Mucilage collected from different plants was pooled and serially diluted from 10^−1^ to 10^−4^. To capture a diversity of bacteria from the rehydrated mucilage, we used a variety of cultivation media (Table 1). To select for anaerobic bacteria, plates were placed in anaerobic jars containing three bags of anaerobic gas generator (Mitsubishi AnaeroPack System). All plates were incubated at 25°C and 37°C for up to 14 days. Isolated colonies were transferred onto new plates with the same medium as used for isolation to confirm purity. Bacteria were grown overnight on R2A broth at 28°C with shaking at 200 rpm, and glycerol stock (25% vol/vol) of pure isolates was stored at −80°C. Prior to performing whole genome sequencing, we performed full-length 16S rRNA gene sequencing, with universal primers 27F (5′-AGAGTTTGATCCTGGCTCAG-3′) and 1492R (5′-TACGGTTACCTTGTTACGACTT-3′) (4), of each isolate using the Sanger protocol to link them to corresponding 16S rRNA gene amplicon dynamics in a field microbiome study that was executed at the same field site at Kellogg Biological Station (3).

**TABLE 1:** Taxonomy, colony phenotype, and genome characteristics of 49 bacterial isolates from sorghum aerial root mucilage, as described in this study^*a*^

Isolate ID	Bacterial species	Initial isolation media	Temperature, oxygen availability	Colony phenotype	Sequencing platform	SRA accession number	GenBank accession	Genome length (bp)	No. of contigs	Coverage	GC%	Genome completeness (%)
SORGH_AS_0335	*Acidovorax wautersii*	R2A	25°C, anaerobic	Small, white	PacBio	SRP437222	JAVIZX000000000	4,571,377	1	70.4×	68.30	99.52
SORGH_AS_0440	*Agrobacterium* sp.	M9 + 0.4% sucrose	25°C, aerobic	White	PacBio	SRP437134	JAVIYR000000000	5,277,481	3	166.0×	59.30	99.04
SORGH_AS_0745	*Agrobacterium* sp.	M9 nitrogen-free + 1% xylose	37°C, aerobic	White, EPS production	PacBio	SRP437174	JAUTBI000000000	5,670,289	4	177.6×	58.60	99.88
SORGH_AS_0749	*Agrobacterium tumefaciens*	M9 nitrogen-free, carbon-free	37°C, aerobic	White, EPS production	PacBio	SRP437121	JAURUU000000000	5,669,583	4	151.8×	58.40	99.93
SORGH_AS_0212	*Arthrobacter* sp.	Jensen	25°C, aerobic	White, EPS production	PacBio	SRP437201	JAUTAA000000000	4,369,871	1	117.7×	66.10	99.71
SORGH_AS_0304	*Atlantibacter* sp.	Jensen	25°C, anaerobic	White	PacBio	SRP437126	JAVLWF000000000	4,876,478	2	140.5×	56.20	99.39
SORGH_AS_0510	*Bacillus* sp.	R2A + 0.1% linalool	37°C, aerobic	White	PacBio	SRP437241	JAUTAU000000000	4,828,420	1	171.6×	38.90	98.52
SORGH_AS_0407	*Bacillus thuringiensis*	R2A + PEG6000—0.73 MPa	25°C, aerobic	White	PacBio	SRP437118	JAURUT000000000	5,894,929	5	106.5×	35.40	99.34
SORGH_AS_0431	*Brevundimonas vesicularis*	M9 + 0.4% sucrose	25°C, aerobic	Pink	PacBio	SRP437147	JAUTAQ000000000	3,191,065	1	68.5×	66.20	99.05
SORGH_AS_0447	*Chryseobacterium* sp.	TSA	25°C, aerobic	Yellow	PacBio	SRP437232	JAUTAR000000000	4,374,394	1	132.6×	39.90	99.51
SORGH_AS_0776	*Curtobacterium* sp.	M9 nitrogen-free + 1% xylose	37°C, aerobic	Yellow	PacBio	SRP437163	JAVIYU000000000	3,877,562	1	164.5×	71.30	98.99
SORGH_AS_0287	*Enterobacter* sp.	Jensen	37°C, anaerobic	White, EPS production	PacBio	SRP437233	JAVIZW000000000	4,947,816	2	108.4×	54.60	99.97
SORGH_AS_0622	*Flavobacterium* sp.	MMS methanol	25°C, aerobic	Orange yellow	PacBio	SRP437242	JAUTAX000000000	5,243,061	1	137.1×	33.80	98.94
SORGH_AS_0428	*Microbacterium* sp.	M9 + 0.4% sucrose	25°C, aerobic	Light yellow, EPS production	PacBio	SRP437182	JAVIZT000000000	3,300,177	1	89.6×	69.60	98.23
SORGH_AS_0445	*Microbacterium foliorum*	TSA	25°C, aerobic	Light yellow	PacBio	SRP437225	JAVIZQ000000000	3,850,168	1	187.5×	67.90	99.14
SORGH_AS_0209	*Microbacterium* *proteolyticum*	Jensen	25°C, aerobic	White, EPS production	PacBio	SRP437239	JAUSZZ000000000	3,767,688	1	122.8×	70.20	98.48
SORGH_AS_0344	*Microbacterium* sp.	R2A	37°C, aerobic	Pink	PacBio	SRP437155	JAUTAD000000000	3,750,535	1	78.7×	70.20	97.98
SORGH_AS_0421	*Microbacterium* sp.	M9 + 0.4% sucrose	25°C, aerobic	Orange	PacBio	SRP437226	JAUTAO000000000	3,450,105	1	228.2×	69.50	97.72
SORGH_AS_0454	*Microbacterium* sp.	TSA	25°C, aerobic	Orange	PacBio	SRP437164	JAVIYS000000000	3,569,378	1	197.0×	69.60	97.98
SORGH_AS_0505	*Microbacterium* sp.	M9 nitrogen-free + 1% xylose	25°C, aerobic	Orange, EPS production	PacBio	SRP437119	JAUTAT000000000	2,952,848	1	139.1×	69.60	97.79
SORGH_AS_0426	*Microbacterium testaceum*	M9 + 0.4% sucrose	25°C, aerobic	Orange	PacBio	SRP437123	JAUTAP000000000	3,569,373	1	202.9×	69.80	97.31
SORGH_AS_0594	*Microbacterium testaceum*	Jensen	37°C, aerobic	Orange, EPS production	PacBio	SRP437144	JAUTAW000000000	3,750,737	1	192.6×	70.20	97.98
SORGH_AS_0422	*Mucilaginibacter terrae*	M9 + 0.4% sucrose	25°C, aerobic	Pink, EPS production	PacBio	SRP437139	JAVLVU000000000	5,485,925	1	173.4×	43.00	97.62
SORGH_AS_0306	*Paenibacillus* sp.	Jensen	25°C, anaerobic	White, EPS production	PacBio	SRP437208	JAUTBP000000000	5,288,478	1	208.8×	39.20	99.45
SORGH_AS_0338	*Paenibacillus* sp.	R2A	25°C, anaerobic	Pink, EPS production	PacBio	SRP437186	JAVIYQ000000000	5,288,506	1	244.2×	39.20	99.36
SORGH_AS_0197	*Pantoea ananatis*	Pirovskaya	25°C, aerobic	Yellow	PacBio	SRP437179	JAUTBM000000000	5,000,175	5	197.6×	53.40	99.34
SORGH_AS_0213	*Pantoea ananatis*	Jensen	25°C, aerobic	Yellow, EPS production	Illumina	SRP437485	JAVIZK000000000	4,919,493	39	310.9×	56.50	100
SORGH_AS_0797	*Pantoea anthophila*	M9 nitrogen-free + 1% arabinose	37°C, aerobic	White, EPS production	PacBio	SRP437125	JAUTBH000000000	4,704,313	5	127.5×	56.60	100
SORGH_AS_0585	*Pedobacter agri*	Jensen	25°C, aerobic	Pink	PacBio	SRP437169	JAUTAV000000000	5,055,982	1	181.8×	38.00	97.61
SORGH_AS_0191	*Pseudomonas fluorescens*	KB	25°C, aerobic	White, fluorescens, EPS production	PacBio	SRP437154	JAVIZU000000000	6,080,362	1	43.2×	60.10	99.52
SORGH_AS_0201	*Pseudomonas* *psychrotolerans*	Pirovskaya	25°C, aerobic	Yellow	PacBio	SRP437137	JAVJAF000000000	5,262,371	1	156.0×	64.70	98.47
SORGH_AS_0199	*Pseudomonas* sp.	Pirovskaya	25°C, aerobic	Yellow	PacBio	SRP437202	JAVJAE000000000	5,020,881	1	146.4×	65.90	99.77
SORGH_AS_0211	*Pseudomonas* sp.	Jensen	25°C, aerobic	Yellow	PacBio	SRP437120	JAVIZV000000000	4,840,922	1	62.3×	66.10	99.87
SORGH_AS_0260	*Rhizobium* sp.	M9 + 0.4% sucrose	37°C, anaerobic	White	PacBio	SRP437152	JAURUW000000000	5,274,802	3	126.6×	59.30	99.62
SORGH_AS_0285	*Rhizobium* sp.	Jensen	25°C, aerobic	Yellow	PacBio	SRP437128	JAURUX000000000	5,277,463	3	136.4×	59.30	99.36
SORGH_AS_0755	*Rhizobium* sp.	M9 nitrogen-free, carbon-free	37°C, aerobic	White, EPS production	PacBio	SRP437200	JAURUV000000000	5,423,623	3	122.8×	59.40	99.84
SORGH_AS_0787	*Rhizobium* sp.	M9 nitrogen-free + 1% arabinose	25°C, aerobic	White, EPS production	PacBio	SRP437131	JAUTBG000000000	5,400,756	3	141.4×	58.20	98.19
SORGH_AS_0301	*Rhodococcus* sp.	Jensen	25°C, aerobic	Pink, EPS production	PacBio	SRP437130	JAUTAC000000000	4,603,776	2	55.1×	68.60	97.93
SORGH_AS_0303	*Rhodococcus* sp.	Jensen	25°C, aerobic	Pink, EPS production	PacBio	SRP437177	JAUTBO000000000	4,508,481	1	162.5×	68.50	98.78
SORGH_AS_0500	*Siphonobacter* sp.	M9 nitrogen-free +	25°C, aerobic	Orange, EPS production	PacBio	SRP437204	JAVIYT000000000	6,158,200	9	119.3×	44.10	99.70
SORGH_AS_0438	*Sphingomonas* sp.	M9 + 0.4% sucrose	25°C, aerobic	Orange	PacBio	SRP437229	JAVIZS000000000	4,040,749	4	165.8×	67.80	98.99
SORGH_AS_0742	*Sphingomonas* sp.	M9 nitrogen-free + 1% xylose	37°C, aerobic	Yellow	PacBio	SRP437158	JAVIZP000000000	4,247,105	2	30.2×	67.10	99.25
SORGH_AS_0789	*Sphingomonas* sp.	M9 nitrogen-free + 1% arabinose	37°C, aerobic	Yellow	PacBio	SRP437240	JAVIYV000000000	4,247,089	2	13.5×	67.10	99.42
SORGH_AS_0802	*Sphingomonas* sp.	M9 nitrogen-free + 1% galactose	25°C, aerobic	Orange	Illumina	SRP437478	JAVIZL000000000	3,943,888	20	398.6×	67.10	99.55
SORGH_AS_0457	*Stenotrophomonasrhizophila*	TSA	25°C, aerobic	White	PacBio	SRP437136	JAUTAS000000000	4,160,543	1	107.6×	66.70	99.71
SORGH_AS_0321	*Stenotrophomonas* sp.	Jensen	25°C, anaerobic	Yellow	PacBio	SRP437127	JAVIZG000000000	4,110,757	1	159.9×	66.70	99.20
SORGH_AS_0282	*Stenotrophomonas* sp.	Jensen	37°C, aerobic	Yellow	PacBio	SRP437122	JAUTAB000000000	4,172,479	1	166.1×	66.70	99.77

^*a*^ SRA is the sequence read archive of the U.S. National Institutes of Health.

Individual isolates or the full cryopreserved collection is available. Cultures can be regrown using standard microbiological procedures to transfer a sterile inoculation loop of freezer stock onto the original isolation medium and incubation time (Table 1).

**Fig 1:**
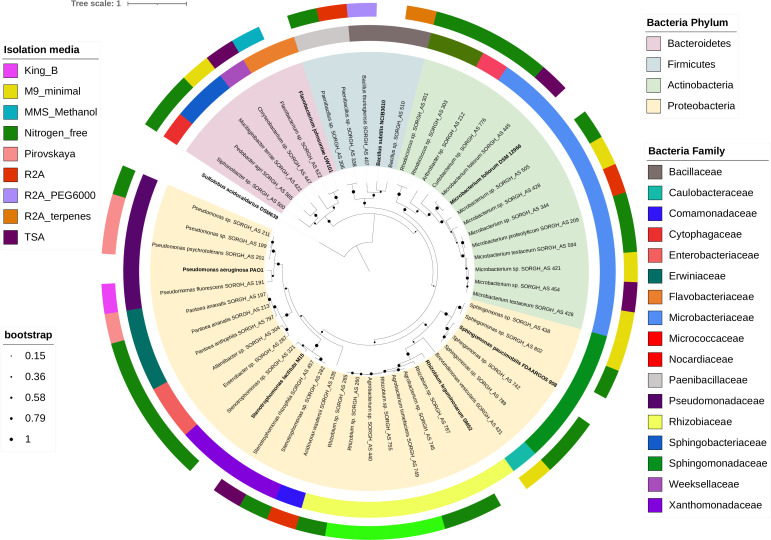
Phylogenetic diversity of the bacterial collection cultivated from sorghum aerial root mucilage. The archaea *Sulfolobus acidocaldarius* DSM 639 (ASM2847236v1) was included as an outgroup. The bacterial reference strains *Pseudomonas aeruginosa* PA01 (ASM676v1), *Microbacterium foliorum* DSM 12966 (ASM95641v1), *Stenotrophomonas lactitubi* M15 (ASM280351v1), *Bacillus subtilis* subsp. *subtilis* NCIB 3610 (ASM608879v1), *Flavobacterium johnsoniae* UW101 (ASM1664v1), *Sphingomonas paucimobilis* FDAARGOS_908 (ASM1602709v1), and *Rhizobium leguminosarum* SM52 (ASM430655v1) were also included. The species tree was inferred from unrooted orthogroups gene trees using STAG and then rooted using the STRIDE algorithms implemented in OrthoFinder. Species tree was annotated with iTOLs.

The high molecular weight genomic DNA of each bacterial isolate was extracted using a phenol/chloroform extraction protocol (5). The genomic DNA of each strain was submitted to the U.S. Department of Energy Joint Genome Institute, for library preparation and sequencing with the Pacific Biosciences (PacBio) platform (6). DNA was sheared to 10 kb using g-TUBE columns (Covaris) and subjected to library preparation using the SMARTbell Express Template prep 2.0 kit and sequenced on the PacBio Sequel platform. Samples that failed DNA quality control required by the PacBio pipeline were instead sequenced using the NovaSeq S4 Illumina platform (7). High fidelity CCS reads > 5 kb were assembled with Flye 2.8.3 (8) using default settings. Raw Illumina sequences were quality-filtered using BBTools (9). Artifact filtered and normalized Illumina reads were assembled with SPAdes v3.15.3 (10) (–phred-offset 33 –cov-cutoff auto -t 16 -m 64 –careful -k 25,55,95), and contigs were discarded if the length was <1 kb (BBTools reformat.sh: minlength = 1,000 ow = t). Genome completeness and contamination was estimated with CheckM v1.2.2 (11). The assembly was annotated using Prokka 1.14.6 (12) and the phylogenetic tree was inferred using Orthofinder 2.5.5 (13) and edited with iTOLs v.6.5.8 (14) (Fig. 1).

## Data Availability

Raw sequencing data are available at the Joint Genome Institute’s GOLD database under Study ID Gs0157305, as well as with the National Institutes of Health Sequence Read Archive (see Table 1). Genome sequencing assembly data have been deposited in the GenBank with accession numbers listed in Table 1. The bacterial collection is available at The Great Lakes Bioenergy Research Center at Michigan State University, USA (glbrc@msu.edu).

## References

[1] Galloway AF, Knox P, Krause K. 2020. Sticky mucilages and exudates of plants: putative microenvironmental design elements with biotechnological value. New Phytol 225:1461–1469. doi:10.1111/nph.16144 31454421

[2] Bennett AB, Pankievicz VCS, Ané J-M. 2020. A model for nitrogen fixation in cereal crops. Trends Plant Sci 25:226–235. doi:10.1016/j.tplants.2019.12.004 31954615

[3] Mechan-Llontop ME, Mullet J, Shade A. 2023. Phyllosphere exudates select for distinct microbiome members in sorghum epicuticular wax and aerial root mucilage. Phytobiomes Journal 7:184–197. doi:10.1094/PBIOMES-08-22-0046-FI

[4] Miller CS, Handley KM, Wrighton KC, Frischkorn KR, Thomas BC, Banfield JF. 2013. Short-read assembly of full-length 16S amplicons reveals bacterial diversity in subsurface sediments. PLoS One 8:e56018. doi:10.1371/journal.pone.0056018 23405248 PMC3566076

[5] Kutchma AJ, Roberts MA, Knaebel DB, Crawford DL. 1998. Small-scale isolation of genomic DNA from Streptomyces mycelia or spores. Biotechniques 24:452–456. doi:10.2144/98243st05 9526657

[6] Eid J, Fehr A, Gray J, Luong K, Lyle J, Otto G, Peluso P, Rank D, Baybayan P, Bettman B, Bibillo A, et al.. 2009. Real-time DNA sequencing from single polymerase molecules. Science 323:133–138. doi:10.1126/science.1162986 19023044

[7] Bennett S. 2004. Solexa Ltd. Pharmacogenomics 5:433–438. doi:10.1517/14622416.5.4.433 15165179

[8] Kolmogorov M, Yuan J, Lin Y, Pevzner PA. 2019. Assembly of long, error-prone reads using repeat graphs. Nat Biotechnol 37:540–546. doi:10.1038/s41587-019-0072-8 30936562

[9] Bushnell B. 2014. BBtools software package. https://sourceforge.net/projects/bbmap/.

[10] Bankevich A, Nurk S, Antipov D, Gurevich AA, Dvorkin M, Kulikov AS, Lesin VM, Nikolenko SI, Pham S, Prjibelski AD, Pyshkin AV, Sirotkin AV, Vyahhi N, Tesler G, Alekseyev MA, Pevzner PA. 2012. SPAdes: a new genome assembly algorithm and its applications to single-cell sequencing. J Comput Biol 19:455–477. doi:10.1089/cmb.2012.0021 22506599 PMC3342519

[11] Parks DH, Imelfort M, Skennerton CT, Hugenholtz P, Tyson GW. 2015. CheckM: assessing the quality of microbial genomes recovered from isolates, single cells, and metagenomes. Genome Res 25:1043–1055. doi:10.1101/gr.186072.114 25977477 PMC4484387

[12] Seemann T. 2014. Prokka: rapid prokaryotic genome annotation. Bioinformatics 30:2068–2069. doi:10.1093/bioinformatics/btu153 24642063

[13] Emms DM, Kelly S. 2019. OrthoFinder: phylogenetic orthology inference for comparative genomics. Genome Biol 20:238. doi:10.1186/s13059-019-1832-y 31727128 PMC6857279

[14] Letunic I, Bork P. 2021. Interactive tree of life (iTOL) V5: an online tool for phylogenetic tree display and annotation. Nucleic Acids Res 49:W293–W296. doi:10.1093/nar/gkab301 33885785 PMC8265157

