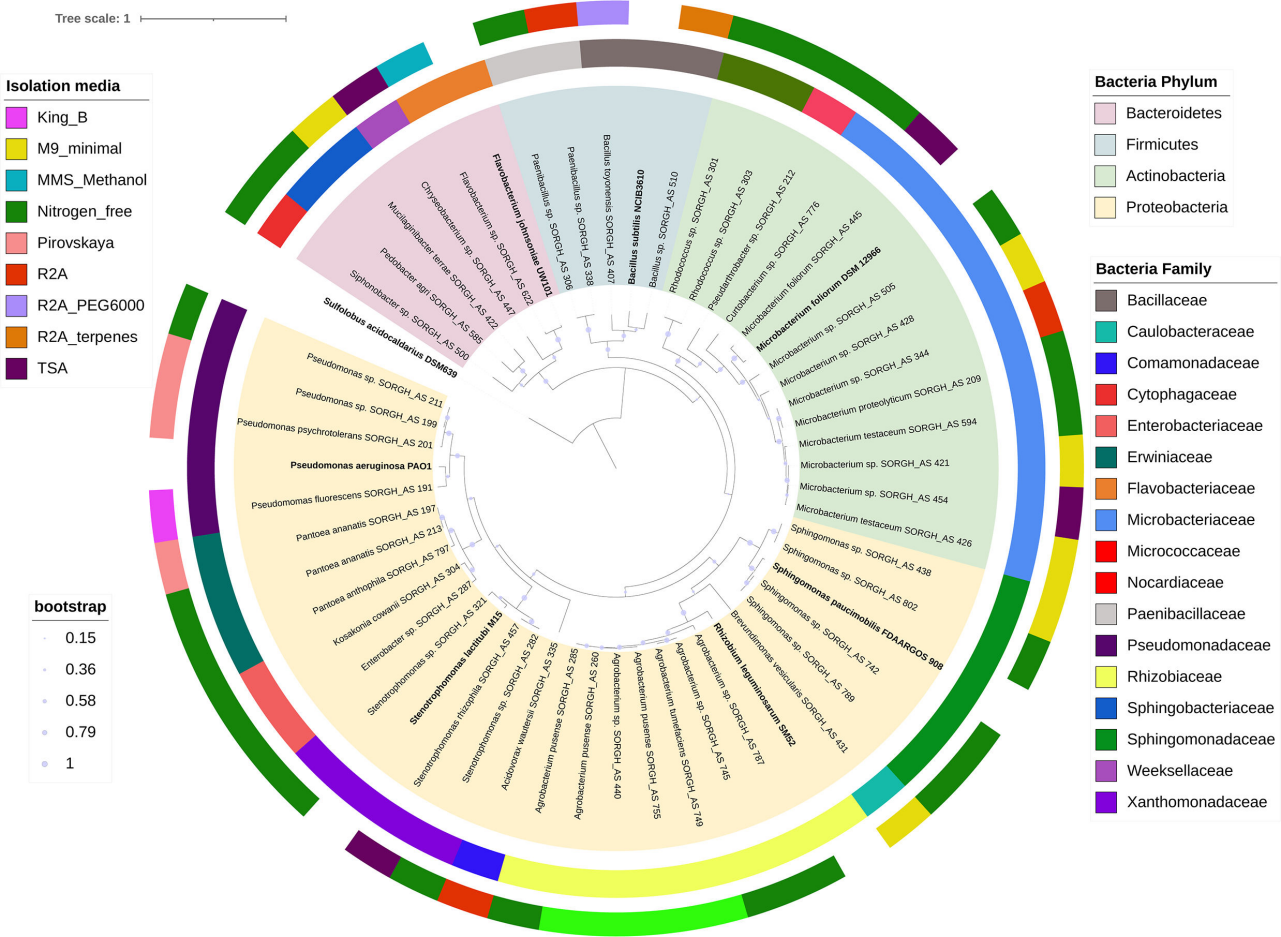# Correction for Mechan-Llontop et al., “Genome-sequenced bacterial collection from sorghum aerial root mucilage”

**DOI:** 10.1128/mra.00521-24

**Published:** 2024-07-01

**Authors:** Marco E. Mechan-Llontop, John Mullet, Ashley Shade

## AUTHOR CORRECTION

Volume 12, no. 12, e00468-23, 2023, https://journals.asm.org/doi/10.1128/mra.00468-23. Page 2, Table 1: Isolate ID SORGH_AS_0212 identified as “*Arthrobacter* sp.*”* should read “*Pseudarthrobacter* sp.”; isolate ID SORGH_AS_0304 identified as “*Atlantibacter* sp.*”* should read “*Kosakonia cowanii*”; isolate ID SORGH_AS_0407 identified as “*Bacillus thuringiensis”* should read “*Bacillus toyonensis*.”

Page 3, Table 1: Isolate ID SORGH_AS_0260 identified as “*Rhizobium* sp.*”* should read “*Agrobacterium pusense*”; isolate ID SORGH_AS_0285 identified as “*Rhizobium* sp.*”* should read “*Agrobacterium pusense*”; isolate ID SORGH_AS_0755 identified as “*Rhizobium* sp.*”* should read “*Agrobacterium pusense*”; isolate ID SORGH_AS_0787 identified as “*Rhizobium* sp.*”* should read “*Agrobacterium* sp.”

Page 5: Figure 1 should appear as shown in this correction.

**Fig 1:**